# Rapid detection of human mastadenovirus species B by recombinase polymerase amplification assay

**DOI:** 10.1186/s12866-018-1365-7

**Published:** 2019-01-08

**Authors:** Tao Wu, Haizhen Wu, Kangchen Zhao, Chaoyou Hu, Yiyue Ge, Xiaojuan Zhu, Xingchen Zhang, Minghao Zhou, Fengcai Zhu, Lunbiao Cui

**Affiliations:** 1 Institute of Pathogenic Microbiology, Key Laboratories of Enteric Pathogenic Microbiology (Ministry of Health), Jiangsu Provincial Center for Disease Control and Prevention, Nanjing, 210009 China; 2 Kunshan Municipal Center for Disease Control and Prevention, Kunshan, 215300 China; 3 Key Laboratory of Infectious Diseases, School of Public Health, Nanjing Medical University, Nanjing, 210029 China; 4 Jiangsu Provincial Center for Disease Prevention and Control, 172 JiangSu Road, Nanjing, 210009 China

**Keywords:** Human mastadenoviruses, Recombinase polymerase amplification, Real-time, Lateral-flow device

## Abstract

**Background:**

As an important component of the causative agent of respiratory tract infections, enteric and eye infections, Human mastadenoviruses (HAdVs) species B spread easily in the crowd. In this study, we developed a recombinase polymerase amplification (RPA) assay for rapidly detecting HAdVs species B which was comprised of two different formats (real-time and lateral-flow device).

**Results:**

This assay was confirmed to be able to detect 5 different HAdVs species B subtypes (HAdV-B3, HAdV-B7, HAdV-B11, HAdV-B14 and HAdV-B55) without cross-reactions with other subtypes and other respiratory tract pathogens. This RPA assay has not only highly sensitivity with low detection limit of 50 copies per reaction but also short reaction time (< 15 min per detection). Furthermore, the real-time RPA assay has excellent correlation with real-time PCR assay for detection of HAdVs species B presented in clinical samples.

**Conclusions:**

Thus, the RPA assay developed in this study provides an effective and portable approach for the rapid detection of HAdVs species B.

## Background

Human mastadenoviruses (HAdVs) are an important cause of human disease, most of which were associated with upper and lower respiratory tract infections, enteric or eye infections. In addition, hepatitis, meningoencephalitis, cystitis and myocarditis, even noninflammatory conditions, such as obesity were also found to be clinical manifestations of HAdVs infections [1–3]. Recently, the putative oncogenicity of certain types of the HAdVs in human was proposed based on the results from mammalian animal models and human malignant diseases [4–6]. So far, there have already been identified out more than 60 serotypes of AdVs in the world [7], about one-third of which were confirmed to be associated with human infections [8]. HAdVs infection happened not only in children but also in adults. But more than 80% of infections of HAdVs occured in children, especially those younger than 4 years old [9]. HAdVs are divided into seven species (HAdV-A to G) on the basis of DNA genome homology [7]. The serotypes most frequently associated with respiratory infection are belonged to species B, species C and species E. Some of HAdV-B and HAdV-C, such as HAdV-B3, HAdV-B7, HAdV-B11, HAdV-B14, HAdV-B55 and HAdV-C2 are common causative agents of respiratory tract infections in children [1, 10]. HAdV-F, such as HAdV-F41, is frequently implicated as a cause of infectious diarrhea worldwide [11].

HAdVs infections are easily spread among the crowd. Timely diagnosis of HAdVs infections will undoubtedly help to prevent theirs transmission. Although viral isolation was the gold standard to identify adenoviral infection, it was time-consuming and needed skilled performers. On the other hand, immunological assay, such as direct immunofluorescence, was rapid to detect viruses. However, this assay usually did not show enough sensitivity and needed special antiserum. With the progress in molecular detection techniques, PCR or real-time PCR assays have rendered rapid and sensitive tools for detection, typing, and monitoring of adenoviral infections [12–15]. However, field application was impeded in resource-limited setting due to potential contamination and needing expensive equipment. Novel isothermal amplification techniques provided substituted tools for HAdVs detection obviating the need for a thermal cycler. Several isothermal amplification assays for detecting HAdVs have been developed [16–18]. All of these assays adopted loop-mediated isothermal amplification (LAMP) methods. In this study, we present a recently developed isothermal amplification method, named recombinase polymerase amplification (RPA), for detecting HAdVs species B. RPA was the proprietary technology of TwistDx Inc.UK. The RPA reaction exploits recombinase, single-stranded DNA binding (SSB) protein and polymerase to initiate the amplification reaction at low temperature (around 37 °C) within minutes. Three TwistAmp amplification formulations were supplied by the provider. TwistAmp basic kit contains the above basic components for the amplification. Amplicons will typically be assessed by an endpoint method, such as gel electrophoresis. TwistAmp exo kit which contains a powerful nuclease (Exonuclease III) can process TwistAmp exo probes during the amplification reaction itself and generate a real-time readout. TwistAmp nfo kit which includes a nfo nuclease (Endonuclease IV) can generate new polymerase extension substrates with suitable nof probes. The antigenic label on the 5′ end of the probe (typically 6-Carboxyfluorescein, FAM or fluorescein isothiocyanate, FITC) becomes conjoined with an antigenic label on the 5′ end of the opposing amplification primer (typically biotin or DIG) and this association can be detected in a ‘sandwich’ assay such as lateral-flow (LF) strips. To resolve the need for resource-limited settings, portable real-time fluorescence scanner device and lateral flow device (LFD) were applied to detect the RPA products in present study.

## Methods

### Viral isolates and clinical specimens

The six virus strains of HAdVs (HAdV-C2, HAdV-B3, HAdV-B7, HAdV-B11, HAdV-B14, and HAdV-B55) isolated from patient’s samples in our institute using Hep-2 cells. The cells were grown in RPMI-1640 (Gabico, USA) supplemented with 10% fetal bovine serum (FBS) (Gabico, USA) and 1% penicillin/streptomycin (Gabico, USA). The cells were cultured at 37 °C in a humidified atmosphere of air containing 5% CO_2_. Other isolates (positive for the following pathogens including *legionella pneumophila*, *streptococcus pneumoniae*, *neisseria meningitides* and *haemophilus influenza*) and clinical specimens (positive for the following pathogens including HAdV-F41, *mycoplasma pneumoniae*, *chlamydia pneumoniae*, *human bocavirus, and herpes simplex virus*) identified by pathogen specific real-time PCR assays in our institute were used to evaluate the specificity. The clinical specimens, a total of 186 pharyngeal swabs were obtained from the children who suffered from acute respiratory infection within 3 days after the onset of clinical symptoms in 2013–2016. All these isolates and samples were stored at − 70 °C until used for further detection and analysis.

### Primers and probes design and screen

Oligonucleotide primers used in the RPA assays were manually designed based on the conserved region of the hexon gene of HAdVs species B according to the instruction manual provided by TwistDx (Cambridge, United Kingdom), the manufacturer of the RPA reaction. Five sets of primers for candidates were designed and pairs were screened by observing their performance on a 2% agarose gel. After the outer primer pairs were determined, internal probes were screened through a similar process. The set of primers and probe with the brightest band on the agarose gel were chosen for further test.

To generate a detectable product on lateral flow strips, the 5′ end of the forward primer was labeled with a 5′-biotin. The nfo probe was labeled on its 5′ end with a FITC group, a C3 spacer (SpC3) on the 3′ end and a tetrahydrofuran residue (THF) which replaces an internal base. For real-time RPA detection, same primers were used except for without of biotinylation at the 5′ end of the forward primer. Exo probe was conjugated with the FAM and the black hole quencher (BHQ) to the T-bases at internal positions with a THF located in the central part of the two fluorescent groups and a SpC3 labeled on the 3′ end. All oligonucleotides were synthesized by Sangon Biotech (Shanghai, China).

### Preparation of plasmid and DNA extraction

PCR product of the hexon gene sequence was cloned into a pMD19-T vector. The plasmid was extracted from culture using the TaKaRa MiniBEST Plasmid Purification Kit (TaKaRa, China) following the manufacturer’s instruction. The concentration was measured by spectrophotometry at 260 nm. The expected copy number of the target gene was calculated according to a previously described formula [19]. The purified plasmid was used as a template to optimize the RPA assays. Genomic DNA from multiple strains and clinical samples was extracted from samples using QIAamp® MinElute® Virus Spin Kit (Qiagen) according to the manufacturer’s instruction and eluted in a final volume of 50 μl. Extracted DNA was stored at − 70 °C until further use.

### Real-time PCR

Real-time PCR assay for HAdVs was adapted from previous published procedure with slight modifications [13]. In brief, real-time PCR reactions were carried out by using Premix Ex Taq Kit (TaKaRa, China). Twenty microliter reaction volume contained 10 μl of 2 × PCR Buffer, 200 nM of primer mix, 100 nM of probe mix, and 5 μl of extracted DNA. Real-time PCR cycling was performed on LightCycler Real-Time PCR system (Roche) as follows: a 30 s heat activation of the Taq polymerase at 95 °C followed by 40 amplification cycles of 5 s at 95 °C and 20 s at 56 °C each (annealing-extension step).

### LFD-RPA assay

LFD-RPA assay was performed in a 50 μl volume with the TwistAmp nfo kit (TwistDx, Cambridge, UK) containing 420 nM each primer, 120 nM target-specific RPA nfo probe, and 1x rehydration buffer. After mixing these components with dry reagent pellet, 5 μl of DNA was added to the reaction mixture, and 2.5 μl of magnesium acetate (Mg(OAc)_2_, 280 mM) was pipetted into the tube lids. The lids were closed and the magnesium acetate was spun down into the reaction mixture to trigger the reaction. The reaction mix was placed into the heating block with brief mixing and centrifugation after 3–4 min of incubation. Three temperatures (37, 39, and 42 °C) and six reaction times (5, 10, 15, 20 25 and 30 min) were tested during RPA assay. RPA products were detected using LFD (Ustar Biotech Co. Ltd. Hangzhou, China) according to previous procedure [20]. Briefly, the reaction tube was placed into cartridge and was immobilized by closing the cartridge. Then the cartridge was inserted into detection chamber whose handle was then closed to cut open running buffer reservior and reaction tube. Finally the detection result was read by naked eyes after 5 to 10 min.

### Real-time RPA assay

Real-time RPA reaction was set up similar to that of LFD-RPA assay except for using a TwistAmp Exo kit (TwistDX, Cambridge, UK), primers without biotinylation, and nfo probe. The reaction tubes were mixed, centrifuged and then placed into RAA isothermal amplification and detection device (Qitian Co, Wuxi, China). The reaction was performed at 39 °C for 30 min, with brief mixing and centrifugation of reaction tubes after 3–4 min of incubation.

### Sensitivity and specificity of the RPA reactions

The sensitivity of the assay was evaluated using 10-fold serial dilutions of plasmid standards (ranging from 1 × 10^0^–1 × 10^4^copies/μl). The specificity of the RPA was determined by analyzing different strains or clinical specimens positive for the pathogens including differential types of HAdVs (HAdV-C2, HAdV-F41), *mycoplasma pneumoniae*, *chlamydia pneumoniae*, *legionella pneumophila*, *streptococcus pneumoniae*, *neisseria meningitides*, *haemophilus influenza* and *herpes simplex virus*.

### Clinical sample analysis and sequencing

To evaluate the performance characteristics of the real-time RPA assay in clinical sample detection, all of the 186 pharyngeal swab specimens were subjected to real-time RPA assay with the parallel analysis by real-time PCR. The positive specimens were further being molecular typing using the protocol described previously [21].

## Results

### Optimization of the RPA conditions

According to the standard mentioned methods, one set of primers, one nfo and one exo probe were selected (Table 1) for further optimization. RPA was conducted in 15 min at 37, 39 and 42 °C using 1000 copies of plasmid DNA respectively. There were no significant differences in the LFD-RPA and real-time RPA results for amplification at different temperature. Therefore, the temperature at 39 °C was chosen for the subsequent RPA assays. Six different times comprising of 5, 10, 15, 20, 25 and 30 min were used in the LFD-RPA reaction. A signal on the lateral flow strip can be detected after 5 min of amplification reaction at 39 °C. The intensity of RPA amplicons at 10, 15, 20 and 25 min showed no significant differences. However, nonspecific band occurred in the negative controls when reaction time reaching 30 min. To avoid false positive result, 20 min was selected as an optimal reaction time for RPA assay.

**Table 1:** List of primers and probe for the lateral-flow strip and real-time RPA assay based on the HAdVs strain accession no. KX289874

Assay format	Oligo name	Sequence 5′-3’	Genome position
LFD-RPA	ADV-RF-Bio	Bio-GCTCATTGGAACAACTGCCGTAAATAGTGT	20,761–20,790
	ADV-RR	GGCCGAGAACGGTGTACGCAGGTAGACTGTC	21,055–21,025
	ADV-RP-nfo	FITC-CTCGATGACGCCGCGGTGTGGCTGGTGCAC[THF]CTGACCACGTCGAAGA-SpC3	20,979–21,025
Real-time RPA	ADV-RF	GCTCATTGGAACAACTGCCGTAAATAGTGT	20,761–20,790
	ADV-RR	GGCCGAGAACGGTGTACGCAGGTAGACTGTC	21,055–21,025
	ADV-RP-exo	CAGGTAGACTGTCTCGATGACGCCGCGGTGT[FAM]G[THF]CT[BHQ]GGTGCACGCTGACC-SpC3	20,989–21,037

*Bio* Biotin, *FITC* fluorescein isothiocyanate, *THF* tetrahydrofuran, *SpC3* C3 spacer, *FAM* 6-Carboxyfluorescein, *BHQ* black hole quencher

### Analytical sensitivity of the RPA assay

**Fig. 1:**
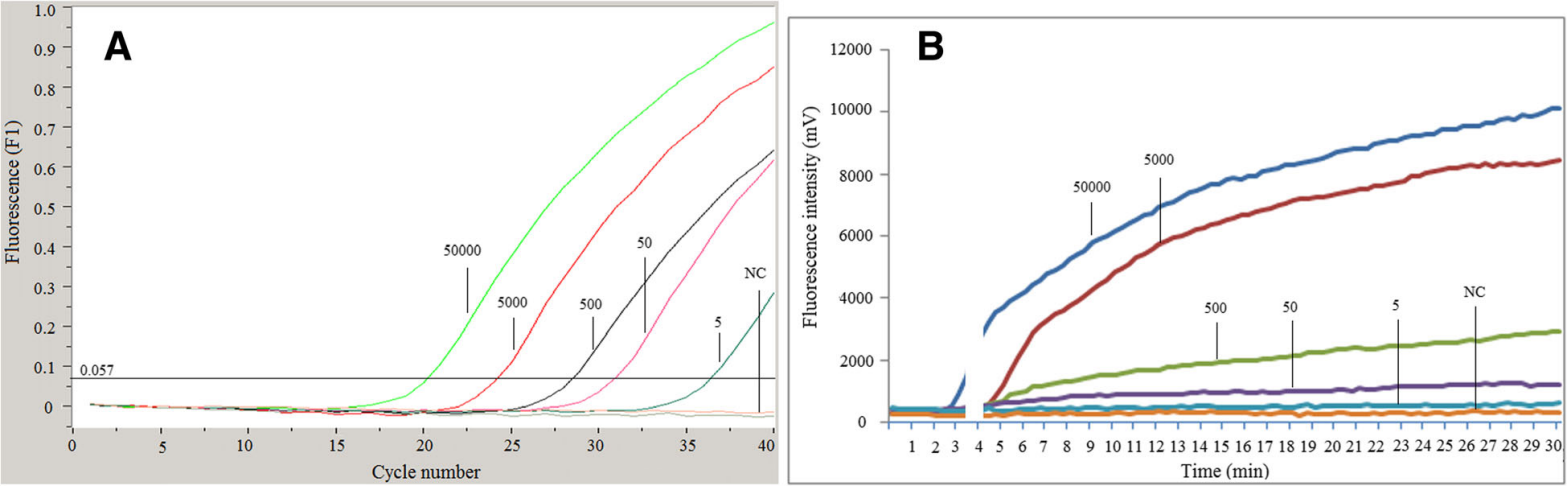
Amplification plots of real-time measurements for 10-fold serial dilutions of plasmid. **a**: real-time PCR results; **b**: real-time RPA results

The detection threshold of the RPA was determined by using a diluted series of plasmid DNA ranging from 5 to 50,000 copies per reaction. The amplified products were analyzed by LFD and real-time fluorescence monitor. Repeatability of the method was assessed by testing each dilution 5 times. The results were compared with that of real-time PCR. Real-time PCR and LFD-RPA assays detected as low as 5 copies per reaction while real-time RPA assay could detect as low as 50 copies per reaction (Table 2 and Fig. 1).

**Table 2:** Sensitivity testing of the real-time and LFD-RPA with a dilution series of plasmid DNA in comparison with real-time PCR results

Template copy number	Real-time PCR	Real-time RPA		LFD-RPA
	Ct Value	Pos. reactions/No. of runs	Means Tt value (min)	Pos. reactions/No. of runs
50,000	20.2	5/5	3.2	5/5
5000	24.1	5/5	5.2	5/5
500	27.7	5/5	5.4	5/5
50	30.8	5/5	5.5	5/5
5	36.1	2/5	6.2	5/5
Neg. control	N.D.	0/5	N.D.	0/5

*Ct* cycle threshold, *Tt* time threshold, *Neg.* Negative, *Pos.* Positive, *N.D*. None determined

### Analytical specificity of RPA assay

**Fig. 2:**
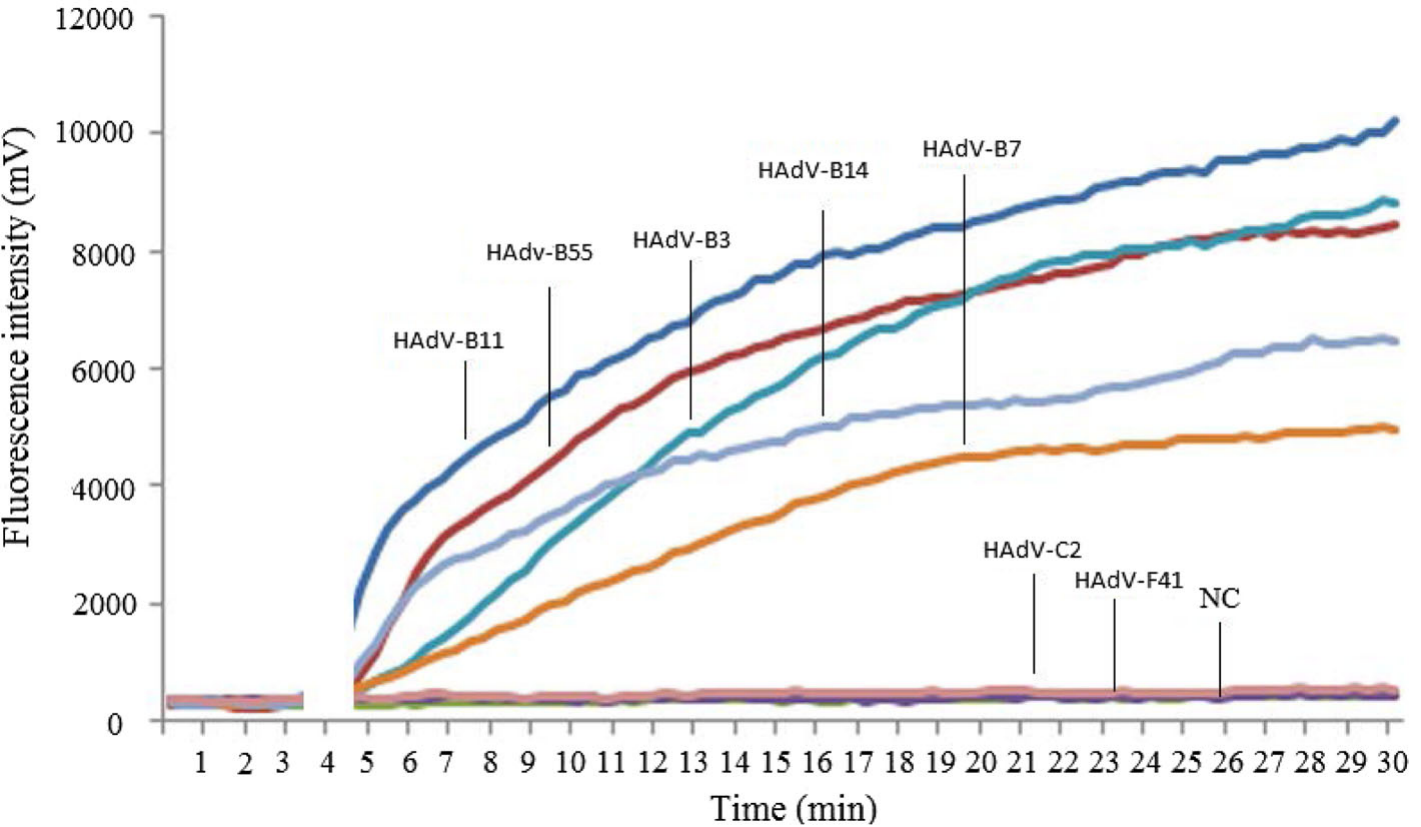
Specificity testing of the real-time RPA with 7 isolates of HAdVs including type HAdV-C2, HAdV-B3, HAdV-B7, HAdV-B11, HAdV-B14, HAdV-F41 and HAdV-B55

The analytical specificity of the RPA assay was evaluated by testing DNA extracts from various pathogens that could cause similar respiratory symptoms. The results showed that the positive reactions were only observed in the extracts of HAdVs species B while testing results for all other pathogens including *mycoplasma pneumoniae, chlamydia pneumoniae, legionella pneumophila, streptococcus pneumoniae, neisseria meningitides, haemophilus influenza, human bocavirus* and *herpes simplex virus* were negative. These results indicate that the analytical specificity of the RPA assay was 100%. To evaluate the applicability of RPA assay to detect HAdVs, seven isolates of HAdVs including five species B (HAdV-B3, HAdV-B7, HAdV-B11, HAdV-B14 and HAdV-B55), one species C (HAdV-C2),one species F (HAdV-F41) were tested. The results revealed HAdVs species B could be detected by both LFD RPA and real-time RPA assays while other species could not be detected (Fig. 2).

### Comparison of real-time RPA assay and real-time PCR to detect HAdVs

Using a total of 186 pharyngeal swabs collected from the children who suffered from acute respiratory infection, the clinical performance characteristics of the real-time RPA assay were assessed with using real-time PCR as reference. As showed in Table 3, 35 pharyngeal swabs were detected positive by real-time PCR assay with Ct values ranging from 15.6 to 35.3. Of the 35 samples detected positive in real-time PCR, 33 were detected positive by real-time RPA assay, resulting in a sensitivity of 94.3%. Of the 151 samples tested negative in real-time PCR, all were also tested negative by real-time RPA assay, providing a specificity of 100%. Two samples tested positive by real-time PCR but negative by real-time RPA were identified by sequencing as HAdV-C2. Other 33 samples tested positive by both methods were identified by sequencing contained 22 HAdV-B3, 7 HAdV-B7, 2 HAdV-B11, and 2 HAdV-B55.

**Table 3:** Performance of real-time RPA assay in comparison to thereference method, real-time RT-PCR, for detecting HAdVs in clinical samples

		Real-time PCR		Performance characteristics (%)	
		Positive	Negative	Sensitivity	Specificity
Real-time RPA	Positive	33	0	94.3	100
	Negative	2	151		
	Total (*n* = 186)	35	151		

## Discussion

HAdV species B is one of the most common pathogens associated with acute respiratory infections, particularly in infants and children. It was difficult for clinicians to distinguish HAdVs infections from others, because clinical symptoms caused by HAdVs infections were similar to those caused by other respiratory pathogens. Thus, rapid, sensitive and specific diagnostic assay for detecting HAdVs species B are urgently needed and would play an important role on initial clinical treatment, avoidance of antibiotic misuse, as well as prevention of virus transmission, especially in developing countries. RPA was a new isothermal amplification method with short turnaround time, low temperature, high sensitivity, and specificity which has been used to detect various viruses [22–24]. In this study, we developed a novel molecular method to detect HAdVs species B by combining RPA with real-time fluorescence detection and LFD technique. Since RPA assay can be conducted at a comparatively low constant temperature (from 37 to 42 °C), it could be carried out with a portable real-time fluorescence scanner device without thermal cycler. On the other hand, the read-out can also be observed by naked eyes with LFD which avoids cross-contamination between specimens [20, 25]. These characteristics of RPA assay for HAdVs species B detection developed in this study is suitable for use as a point-of-care molecular diagnostic assay or in low-equipment setting.

Three isothermal amplification methods for the detection of HAdVs were developed based on LAMP technique which can obtain the results within 45 to 60 min [16–18]. Compared with LAMP, RPA assay performed in shorter reaction time. The result can be read out within 30 min (20 min RPA plus 5 min on LFD). LAMP requires four to six primers to initialize reaction, while RPA-LFD assay only requires three primers. At the time this article was written, no specialized software is available for design RPA primers. It could be further simplified RPA primer design if specialized software would be developed in the future. Previous studies have shown the sensitivity of RPA is parallel to that of LAMP assay while its specificity is higher than that of LAMP [26]. The developed LFD-RPA assay has shown high sensitivity for detection of HAdVs (5 copies/reaction) which is equal to real-time PCR assay, while higher than LAMP assay (50 to 10,000 copies/reaction) [17, 18]. Although the detection limit of real-time RPA is slightly higher than that of LFD-RPA, this level of sensitivity is sufficient to detect HAdVs species B in clinical samples of pediatric patients which the mean virus loads were 1.7 × 10^5^ copies/ml [13]. No positive results were obtained from a large panel of DNA samples from various viruses and bacteria causing respiratory infection. However, we observed nonspecific amplification bands when reaction time exceeded 30 min in LFD-RPA assay. This may due to the clotting of proteins and primers since all tested samples appeared nonspecific bands when reaction time exceeded 30 min. Similar results were also observed in the detection for yellow fever virus [27]. The false-positive results of LFD-RPA limited its field application. So, we only evaluated characteristics of the real-time RPA assay clinically. The results showed real-time RPA assay was able to identify out 94.3% HAdVs positive samples, thereby demonstrating a high degree of specificity. It should be pointed out that this assay could not distinguish different serotypes although 7 serotype HAdVs were tested in this study and could not detect all serotypes. Since there are more than 60 serotypes ADV, it was difficult to design the primers for all serotypes and degenerate primers and probes should be considered to increase its range of application. So far, only one on-chip RPA assay to detect HAdV-F41 was established with the limit of detection of 35 genomic units/μl [28]. Although we successfully identified out the most of clinical samples which were HAdVs positive as confirmed by real-time PCR assay in this study, more samples of other serotypes need to be tested in the future.

In addition, sample preparation techniques capable of extracting DNA in a manner compatible with the RPA assay need to be developed before this assay being used under field conditions. Alkaline lysis and heat treatment seem to be simple and appropriate methods since they have been successfully used in LAMP assay for detection HAdVs [18]. RPA can tolerate inhibitory substances and temperature variations. Whether those methods are fit for RPA assay needs to be tested in the future [29].

## Conclusions

We have developed a very rapid and sensitive isothermal RPA assay for the detection of HAdVs species B infections. This assay has great potential to be a point-of-care diagnostic tool if suitable sample preparation method has been developed.

## Abbreviations

HAdVs: Human mastadenovirus
LAMP: Loop-mediated isothermal amplification
LFD: Lateral flow device
PCR: Polymerase chain reaction
RPA: Recombinase polymerase amplification
